# Inducible Conditional Vascular-Specific Overexpression of Peroxisome Proliferator-Activated Receptor Beta/Delta Leads to Rapid Cardiac Hypertrophy

**DOI:** 10.1155/2016/7631085

**Published:** 2016-03-03

**Authors:** Kay-Dietrich Wagner, Ana Vukolic, Delphine Baudouy, Jean-François Michiels, Nicole Wagner

**Affiliations:** ^1^Institute of Biology Valrose (iBV), University of Nice Sophia Antipolis, CNRS UMR7277/INSERM U1091, Faculty of Medicine, 06107 Nice, France; ^2^Institute for Molecular Health Sciences, ETH Zurich, 8093 Zurich, Switzerland; ^3^Department of Pathology, CHU Nice, 06002 Nice, France

## Abstract

Peroxisome proliferator-activated receptors are nuclear receptors which function as ligand-activated transcription factors. Among them, peroxisome proliferator-activated receptor beta/delta (PPAR*β*/*δ*) is highly expressed in the heart and thought to have cardioprotective functions due to its beneficial effects in metabolic syndrome. As we already showed that PPAR*β*/*δ* activation resulted in an enhanced cardiac angiogenesis and growth without impairment of heart function, we were interested to determine the effects of a specific activation of PPAR*β*/*δ* in the vasculature on cardiac performance under normal and in chronic ischemic heart disease conditions. We analyzed the effects of a specific PPAR*β*/*δ* overexpression in endothelial cells on the heart using an inducible conditional vascular-specific mouse model. We demonstrate that vessel-specific overexpression of PPAR*β*/*δ* induces rapid cardiac angiogenesis and growth with an increase in cardiomyocyte size. Upon myocardial infarction, vascular overexpression of PPAR*β*/*δ*, despite the enhanced cardiac vessel formation, does not protect against chronic ischemic injury. Our results suggest that the proper balance of PPAR*β*/*δ* activation in the different cardiac cell types is required to obtain beneficial effects on the outcome in chronic ischemic heart disease.

## 1. Introduction

Peroxisome proliferator-activated receptors (PPARs) are ligand-activated transcription factors that belong to the nuclear receptor superfamily. There are three members of the PPAR family (*α*, *β*/*δ*, and *γ*) with distinct, but overlapping spatial, temporal, and regulated expression patterns. For all PPARs, lipids are endogenous ligands and PPARs are considered as important transcriptional regulators of genes involved in lipid metabolism and cardiac energy production [1].

PPAR*β*/*δ* is the predominant subtype in the heart, and several lines of evidence suggest a cardioprotective function of PPAR*β*/*δ*. Cardiac PPAR*β*/*δ* deletion in mice resulted in cardiac dysfunction, hypertrophy, and congestive heart failure [2]. Furthermore, it has been shown that the PPAR*β*/*δ* agonist L-165041 inhibits pharmacologically induced hypertrophy of cardiomyocytes through the interaction of PPAR*β*/*δ* to NF-*κ*B and a subsequent downregulation of NF-*κ*B target genes [3, 4]. An* in vivo* study demonstrated that cardiac specific overexpression of PPAR*β*/*δ* led to increased myocardial glucose utilisation and did not alter cardiac function but tended to exert a protective effect to ischemia/reperfusion-induced myocardial injury. This was attributed to an activation of the Glut-4 promoter by PPAR*β*/*δ* and the subsequently increased cardiac glucose utilisation [5]. Finally, we recently showed that pharmacological activation of PPAR*β*/*δ* with GW0742 or GW501516 in mice led to rapid cardiomyocyte growth with a preserved myocardial function. We demonstrated that PPAR*β*/*δ* directly activates the Calcineurin gene [6], which is known to induce cardiac growth [7, 8]. Most interestingly, we observed in our study a rapid induction of cardiac angiogenesis upon pharmacological PPAR*β*/*δ* activation, a matter which surprisingly had not been investigated before, although the correlation between cardiac growth and angiogenesis seems quite evident. PPAR*β*/*δ* expression in endothelial cells has already been reported in 1999 by Bishop-Bailey and Hla [9]. Pharmacological activation of endothelial and endothelial progenitor cells with PPAR*β*/*δ* agonists had been shown to increase the migration, proliferation, and tube formation of these cells [10, 11].

Furthermore, PPAR*β*/*δ* knockout mice exhibited a diminished blood flow and immature microvascular structures in subcutaneously induced tumors, which could be rescued by reexpression of PPAR*β*/*δ* [12]. In human pancreatic tumors, PPAR*β*/*δ* expression strongly correlated with the advanced tumor stage and increased risk of tumor recurrence and distant metastasis. PPAR*β*/*δ* has therefore been suggested to be involved in the regulation of the angiogenic switch in tumor progression [13].

PPAR*β*/*δ* is also involved in physiological angiogenesis. As we and others showed, treatment with the PPAR*β*/*δ* agonists GW0742 and GW501516 induced an exercise-like phenotype in the heart. Both agonists induced a surprisingly rapid (after 24 h) remodelling of mouse hearts [6] and skeletal muscle [14] by increasing microvessel densities.

However, until now it was not clear if either the increase of the cardiac vasculature drives the myocardial hypertrophy or the enhanced cardiac angiogenesis might be a potential indirect effect of cardiomyocyte-specific PPAR*β*/*δ* overexpression.

In our present work, we address this question through the generation of transgenic mice with an inducible conditional vascular-specific overexpression of PPAR*β*/*δ* and analyze the normal cardiac phenotype and function as well as function and histology after experimental myocardial infarction.

We show that inducible vessel-specific overexpression of PPAR*β*/*δ* results in a rapid induction of angiogenesis, cardiac hypertrophy, and impairment of cardiac function as reflected by enhanced end-diastolic and end-systolic volumes, reduced fractional shortening, and decreased ejection fractions. Additionally, we demonstrate that, after myocardial infarction, despite the higher collateral vessel formation, the animals with vascular-specific PPAR*β*/*δ* overexpression display bigger infarct lesions, higher cardiac fibrosis, and further reduced cardiac function. This points to a more careful view about the potential benefits of PPAR*β*/*δ* agonists in the treatment of cardiovascular diseases, as the proper balance between cardiomyocytic and vascular PPAR*β*/*δ* seems to be crucial for cardiac health, especially under ischemic conditions.

## 2. Materials and Methods

### 2.1. Animals

All animals were used in accord with local Home Office regulations.* PPARβ/δ-flox* ^*+/*−^ [15] and* Tie2-CreERT2* [16] animals were crossed to generate* Tie2-CreERT2;PPARβ/δ-flox* ^*+/*−^ mice, further referred to as* Tie2-CreERT2;PPARβ/δ*. The* Tie2-*Cre-line was backcrossed four times onto C57BL6. Age- and sex-matched* Tie2-CreERT2;PPARβ*/*δ* animals were injected for one week intraperitoneally either with sunflower oil (vehicle) or Tamoxifen dissolved in sunflower oil in a dose of 33 mg/kg per day [17].* Tie2-CreERT2* animals injected with Tamoxifen served as an additional control. Anaesthetized mice were examined by echocardiography using the iE33 xMATRIX system with a 12 MHz transducer (Philips Healthcare, DA Best, Netherlands). Myocardial infarctions were induced by ligation of the left coronary artery (LAD) as described [18]. Briefly, anaesthetized mice were endotracheally intubated, the skin was incised on the left thorax side, the pectoralis muscles were mobilized, a thoracotomy between the third and fourth rib was performed, and the LAD permanently was closed with a 7-0 suture distal to the left auricle. This resulted in large myocardial infarctions. The thoracotomy and the skin wound were closed with 4-0 sutures and the mice remained intubated until spontaneous respiration was reestablished. Lethality of the procedure was approximately 50% independent of the genotype of the mice.

### 2.2. Genotyping

The genotype of animals was identified by PCR. PCR conditions and primer sequences are available on request.

### 2.3. Tissue Samples, Histology, and Immunohistology

Histology and measurement of cardiomyocyte diameters were performed according to established protocols [19]. Samples from at least five different animals per group (*Tie2-CreERT2;PPARβ/δ* + vehicle,* Tie2-CreERT2* + Tamoxifen, and* Tie2-CreERT2;PPARβ/δ* + Tamoxifen) were analyzed. Investigators were blinded for the genotype of the mice. Three *μ*m paraffin sections were used for histological and immunohistological procedures.

Haematoxylin-Eosin staining was routinely performed on all tissue samples; additionally, sections were stained with Trichrome Masson and Picrosirius red. For PPAR*β*/*δ* and Pecam-1 immunohistology, after heat-mediated antigen retrieval and quenching of endogenous peroxidase activity, the antigen was detected after antibody application Pecam-1 (CD31) (1 : 100, rabbit polyclonal, ab28364, Abcam) or PPAR*β*/*δ* (1 : 100, rabbit polyclonal, ab154395, Abcam) using EnVision*™* Peroxidase/DAB Detection System from Dako (Trappes, France). Sections were counterstained with Hematoxylin (Sigma). Omission of the first antibody served as a negative control. Additionally, some slides were incubated with IgG Isotype Controls (1 : 100, rabbit monoclonal, clone SP137, Abcam). Slides were viewed under an epifluorescence microscope (DMLB, Leica, Germany) connected to a digital camera (Spot RT Slider, Diagnostic Instruments, Scotland).

Area densities for all immunohistological stainings were determined using the ImageJ software. Vessel area density was analyzed on at least five different sections of hearts per mouse.

### 2.4. Real-Time RT-PCR

Total RNA was isolated from hearts and cardiac endothelial cells, sorted with CD31 MicroBeads (Miltenyi Biotec) from the mouse hearts using the Trizol reagent (Invitrogen). The RNA pellet was dissolved in diethyl pyrocarbonate-treated H_2_O. First-strand cDNA synthesis was performed with 0.5 *μ*g of total RNA using oligo(dT) primers and Superscript III reverse transcriptase (Invitrogen). One *μ*L of the reaction product was taken for real-time RT-PCR amplification (ABI Prism 7000, Applied Biosystems) using a commercial SYBR® Green kit (Eurogentec, Angers, France). Primer sequences are available on request. Expression of each gene was normalized to the respective* Gapdh, Actb, and Rplp0* expression.

### 2.5. Statistical Analysis

Data are expressed as means ± SEM. ANOVA with Bonferroni test as* post hoc* test or Mann-Whitney tests was performed as indicated. A *p* value of less than 0.05 was considered statistically significant.

## 3. Results and Discussion

### 3.1. PPAR*β*/*δ* Vascular-Specific Overexpression Rapidly Increases Cardiac Vessel Density

**Figure 1:**
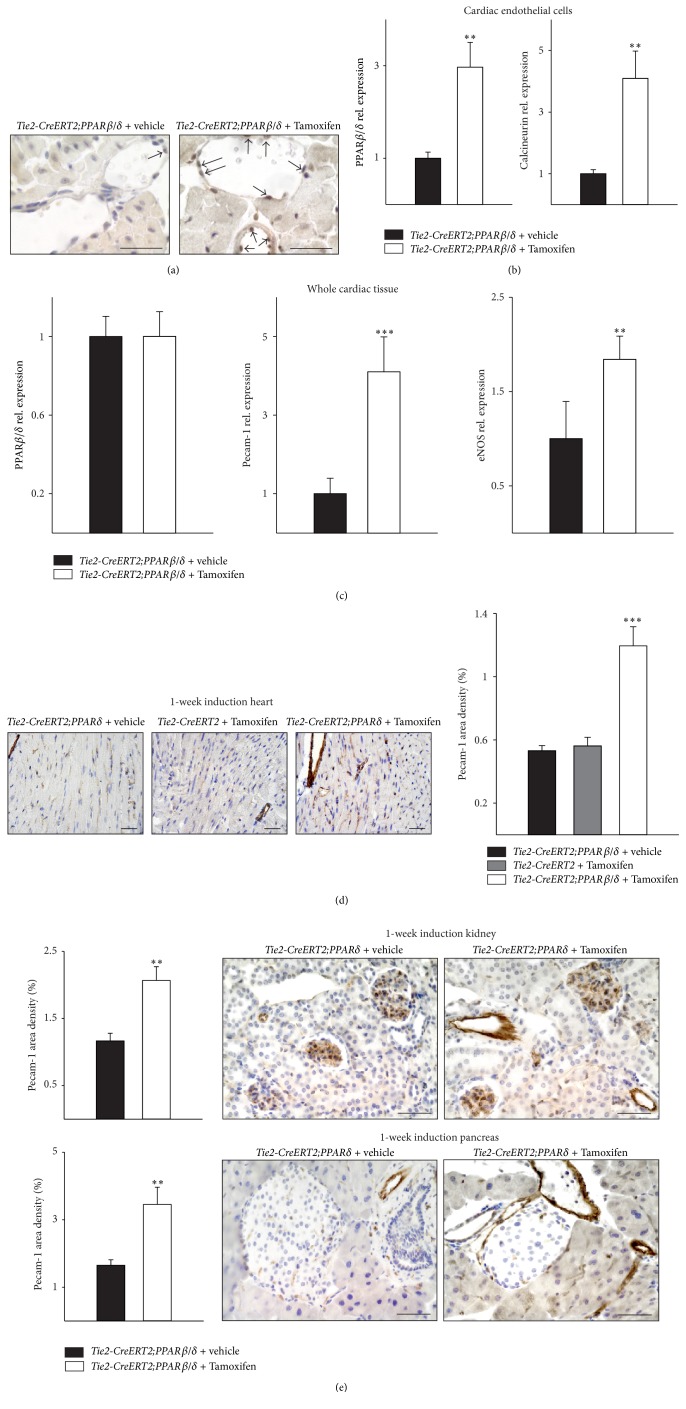
Increased cardiac vessel density upon Tie2-Cre-mediated conditional PPAR*β*/*δ* overexpression. (a) PPAR*β*/*δ* immunostaining from heart sections of* Tie2-CreERT2; PPARβ*/*δ* + vehicle and* Tie2-CreERT2;PPARβ/δ* + Tamoxifen animals indicates higher expression levels in the endothelium of* Tie2-CreERT2;PPARβ/δ* + Tamoxifen animals. Arrows mark PPAR*β*/*δ* positive endothelial cells. (b) Quantitative real-time PCRs for PPAR*β*/*δ* and Calcineurin in cardiac endothelial cells from* Tie2-CreERT2;PPARβ/δ* + vehicle and* Tie2-CreERT2;PPARβ/δ* + Tamoxifen animals (*n* = 5 for each group). (c) Expression levels for PPAR*β*/*δ*, Pecam-1, and eNOS determined by quantitative real-time PCRs from whole mouse heart RNA preparations for both groups (*n* = 5 for each group). (d) Pecam-1-immunostaining in mouse heart sections and quantification of Pecam-1 signal area density (*Tie2-CreERT2;PPARβ/δ* + Tamoxifen, *n* = 5,* Tie2-CreERT2;PPARβ/δ* + vehicle, *n* = 5, and* Tie2-CreERT2* + Tamoxifen, *n* = 5). (e) Quantification of Pecam-1 signal area densities and Pecam-1-immunostainings in mouse kidney (upper panel) and pancreas (lower panel) sections (*Tie2-CreERT2;PPARβ/δ* + Tamoxifen, *n* = 3, and* Tie2-CreERT2;PPARβ/δ* + vehicle, *n* = 3). Scale bars indicate 50 *μ*m. Data are means ± SEM. ^*∗∗*^ *p* < 0.01 and ^*∗∗∗*^ *p* < 0.001.

Immunohistochemistry for PPAR*β*/*δ* of heart sections proved the upregulation of PPAR*β*/*δ* protein expression in the endothelium of* Tie2-CreERT2;PPARβ/δ* mice induced with Tamoxifen as compared to vehicle-treated* Tie2-CreERT2;PPARβ/δ* animals (Figure 1(a)). Quantitative RT-PCRs from cardiac endothelial cells enriched with Pecam-1/CD31 MicroBeads were performed to confirm the vascular overexpression of PPAR*β*/*δ* upon Cre-mediated recombination. Endothelial cells isolated from hearts of Tamoxifen induced* Tie2-CreERT2;PPARδ* animals showed a modest upregulation of PPAR*β*/*δ* and Calcineurin expression compared to cardiac vascular cells of vehicle-treated animals (Figure 1(b)). In contrast, no significant changes in PPAR*β*/*δ* expression levels in whole heart RNA preparations could be detected (Figure 1(c)), additionally confirming specificity of vascular PPAR*β*/*δ* overexpression, as endothelial cells contribute only to around seven percent of the total cell numbers in the mouse heart [20]. An increase in cardiac vessel density became evident on the RNA (Figure 1(c)) as well as on the protein level (Figure 1(d)) already one week after Cre-mediated vascular PPAR*β*/*δ* overexpression. Additionally, increased cardiac eNOS expression confirmed the enhanced cardiac angiogenesis (Figure 1(c)). The detection of Pecam-1 protein expression by immunohistochemistry allowed determining that this upregulation of Pecam-1 was due to the formation of new microvessels (for comparison, see Figure 1(d) right photomicrograph, which depicts higher microvessel formation in the hearts of* Tie2-CreERT2;PPARβ/δ* animals induced with Tamoxifen as compared to vehicle-treated* Tie2-CreERT2;PPARβ/δ* animals on the left or Tamoxifen treated* Tie2-CreERT2* animals in the middle). Determination of Pecam-1 area density indicated a doubling of Pecam-1 positive vascular structures (Figure 1(d)). This angiogenic response to transgenic overexpression of PPAR*β*/*δ* in the endothelium was also observed in the kidney and the pancreas (Figure 1(e)), indicating a general proangiogenic action of PPAR*β*/*δ* in endothelial cells. These findings are in line with previous studies, which reported a rapid enhancement of vessel density upon pharmacological PPAR*β*/*δ* activation [6, 14] and the general view of PPAR*β*/*δ* as a proangiogenic factor [21].

### 3.2. Specific Vascular Overexpression of PPAR*β*/*δ* Induces Cardiac Hypertrophy

**Figure 2:**
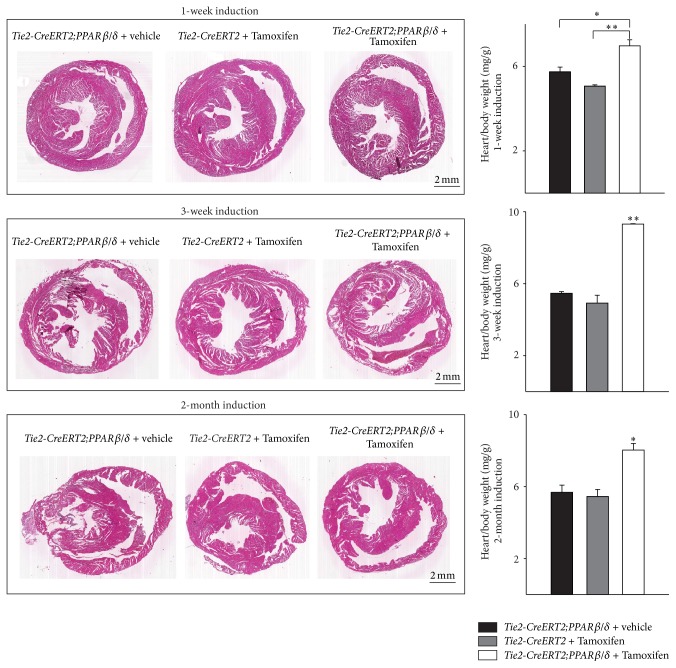
Rapid induction of cardiac growth by Tie2-Cre-mediated conditional PPAR*β*/*δ* overexpression. Photomicrographs of Hematoxylin-Eosin- (HE-) stained cross sections of the hearts and respective heart-to-body weight ratios (*Tie2-CreERT2;PPARβ/δ* + Tamoxifen, *n* = 7,* Tie2-CreERT2;PPARβ/δ* + vehicle, *n* = 6, and* Tie2-CreERT2* + Tamoxifen, *n* = 6). Scale bars indicate 2 mm. Data are means ± SEM. ^*∗*^ *p* < 0.05 and ^*∗∗*^ *p* < 0.01.

**Figure 3:**
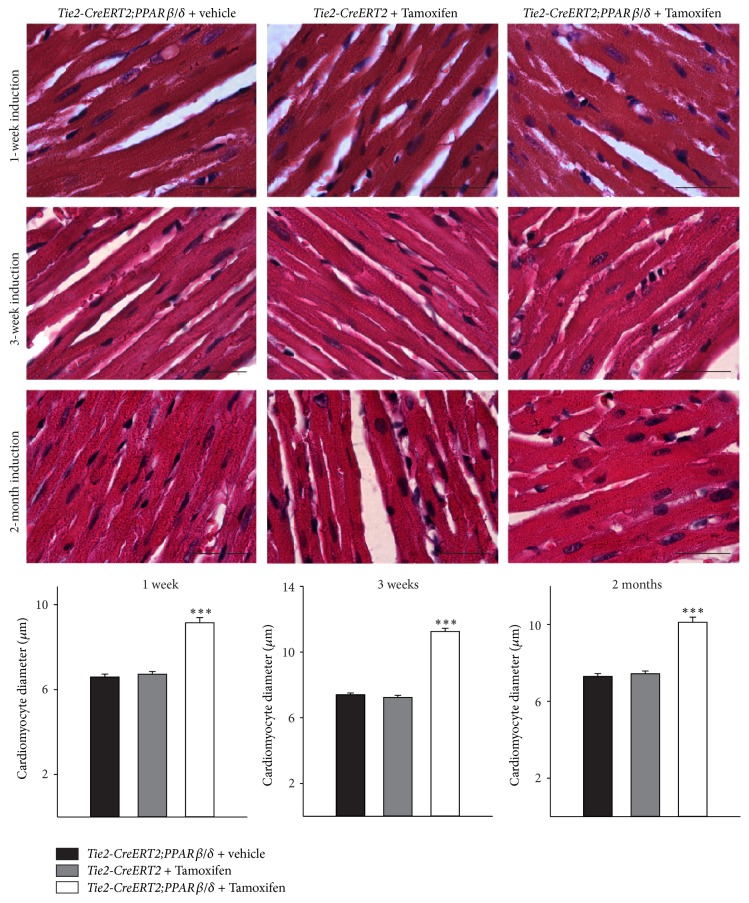
Enhanced cardiomyocyte diameter upon vascular-specific PPAR*β*/*δ* overexpression. High power photomicrographs of HE-stained sections showing individual cardiomyocytes and quantification of cardiomyocyte diameters. Scale bars indicate 50 *μ*m. Data are means ± SEM. ^*∗∗∗*^ *p* < 0.001.

Already one week after induction of PPAR*β*/*δ* expression in vessels, it became evident that cardiac growth was enhanced in the animals with Cre-mediated recombination as compared to both controls, vehicle-treated Tie2-CreERT2;PPAR*β*/*δ* and Tie2-CreERT2 mice treated with Tamoxifen. Heart/body weight measurements confirmed the macroscopic observation. This growth induction became more enhanced after three weeks and remained then stable for up to two months, the latest time point studied (Figure 2). The cause of this cardiac growth was an increase in cardiomyocyte size, as determined by cardiomyocyte diameter measurements at the different time points. On average, the cardiomyocyte diameter increased about 30% compared to the respective controls (Figure 3). Vascular formation during embryonic development is crucial for organ growth; for example, the inhibition of coronary vessel formation abolishes cardiac growth [22]; however, the factors determining organ size in an adult organism are not completely understood, but some lines of evidence suggest that, during tissue repair or in response to physiological stimuli vessel formation is required for organ enlargement [23]. Some evidence that at least for the heart vascular growth indeed led to an increase in the cardiac mass under normal conditions came from a study using transgenic mice with a cardiomyocyte-specific on/off regulatable system for the secretion of the proangiogenic factor PR39. The authors concluded that myocardial hypertrophy observed after three weeks was due to the induction of angiogenesis. They suggested that increased NO production due to increased endothelial cell mass mediated the observed hypertrophy [24]. This is in accordance with our finding of enhanced eNOS expression in the hearts of mice with vascular-specific overexpression of PPAR*β*/*δ* (Figure 1(b)). However, PR39 is a macrophage derived peptide, which inhibits degradation of hypoxia inducible factor 1*α* protein, thus activating angiogenesis through the induction of VEGF and fibroblast growth factor signalling and acting on all cardiac and other cell types. It can therefore not be excluded that part of the observed effects in this study was due to actions of PR39 on other cell types of the heart compared to only endothelial cells. The fact that we could observe cardiac hypertrophy already one week after vascular-specific overexpression of PPAR*β*/*δ* is mostly due to the overexpression of PPAR*β*/*δ* in endothelial cells, which induced angiogenesis leading to hypertrophy of the cardiomyocytes. Our approach was more direct as targeting the secretion of a proangiogenic factor like PR39 by cardiomyocytes, which affects secondarily the endothelium and in the end the increase in cardiomyocyte size is solely attributed to the increased angiogenesis. However, in the mentioned study, it cannot be excluded that the forced secretion of a proangiogenic molecule by cardiomyocytes also acts on other cell types compared to only endothelial cells, including cardiomyocytes themselves. The endothelial-specific conditional induction of PPAR*β*/*δ* in our model excludes a potential interference with possibly in parallel ongoing actions in other cardiac cell types.

### 3.3. PPAR*β*/*δ* Vascular-Specific Overexpression Also Increases Capillary Density in the Setting of Myocardial Infarction but Fails to Ameliorate the Outcome after Chronic Ischemic Heart Disease

**Figure 4:**
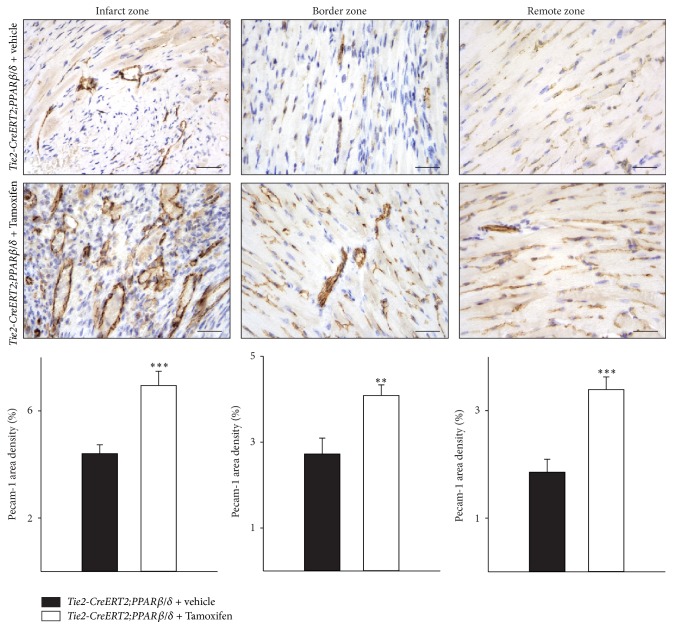
Increased vessel formation after myocardial infarction in the hearts of mice with vascular-specific PPAR*β*/*δ* overexpression. Pecam-1-immunostaining in mouse heart sections and quantification of Pecam-1 signal area density (*Tie2-CreERT2;PPARβ/δ* + Tamoxifen, *n* = 5, and* Tie2-CreERT2;PPARβ/δ* + vehicle, *n* = 5). Scale bars indicate 50 *μ*m. Data are means ± SEM. ^*∗∗*^ *p* < 0.01 and ^*∗∗∗*^ *p* < 0.001.

**Figure 5:**
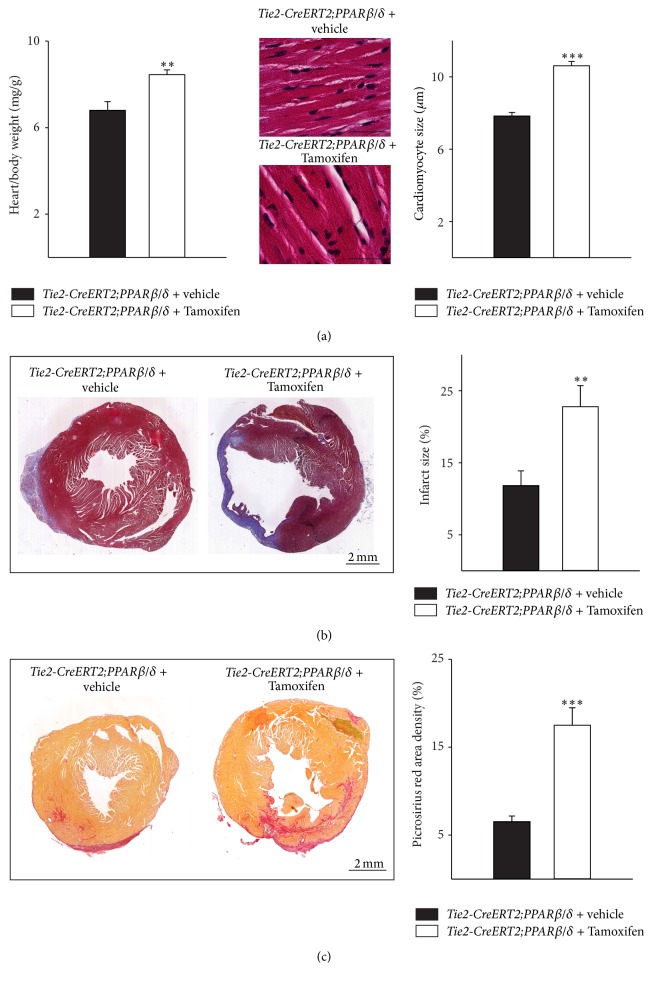
Increased infarct sizes and higher cardiac fibrosis in animals with vascular-specific PPAR*β*/*δ* overexpression. (a) Respective heart-to-body weight ratios and high power photomicrographs of HE-stained heart sections showing individual cardiomyocytes and quantification of cardiomyocyte diameters. Scale bars indicate 50 *μ*m. (b) Photomicrographs of Trichrome Masson stained cross sections and quantification of the infarct sizes (*Tie2-CreERT2;PPARβ/δ* + Tamoxifen, *n* = 8, and* Tie2-CreERT2;PPARβ/δ* + vehicle, *n* = 5). Scale bars indicate 2 mm. (c) Photomicrographs of Picrosirius red stained cross sections and quantification of cardiac fibrosis. Scale bars indicate 2 mm. Data are means ± SEM. ^*∗∗*^ *p* < 0.01 and ^*∗∗∗*^ *p* < 0.001.

To investigate the effect of PPAR*β*/*δ* driven angiogenesis on myocardial function in pathological settings, the left anterior descending (LAD) coronary artery in* Tie2-CreERT2;PPARβ/δ* animals induced with Tamoxifen or treated with vehicle was ligated. Immunohistochemical investigation of Pecam-1 expression demonstrated a significant increase in capillary density not only in the infarct zone but also in the border zone of the infarcted area and in the remote myocardial area of the right ventricle of* Tie2-CreERT2; PPARβ/δ* animals induced with Tamoxifen compared to those treated with vehicle only. This was additionally confirmed by quantification of Pecam-1 area densities (Figure 4). Heart/body weight determination demonstrated a hypertrophic effect of vascular-specific overexpression of PPAR*β*/*δ* also in the setting of chronic ischemic heart disease, due to an increase in cardiomyocyte size (Figure 5(a)). Interestingly, histological analyses revealed much bigger infarct sizes in animals with vascular-specific overexpression of PPAR*β*/*δ* as compared to controls (Figure 5(b)) and an enhanced cardiac fibrosis, as determined by Picrosirius red staining for collagen (Figure 5(c)). This is in contrast to the study using a cardiomyocyte-specific on/off regulatable system for the secretion of the proangiogenic factor PR39 from cardiomyocytes; the secretion of PR39 reduced infarct sizes after myocardial infarction [24]. However, as stated before, this study was based on the effects of PR39, a macrophage derived proangiogenic molecule, which might act on all cardiac cell types rather than solely on endothelial cells. Our results are in agreement with clinical studies suggesting cardiac hypertrophy as a risk factor for arteriosclerosis, myocardial infarction, and heart failure [25]. This is probably due to the increased energy consumption of hypertrophic myocardium.

**Figure 6:**
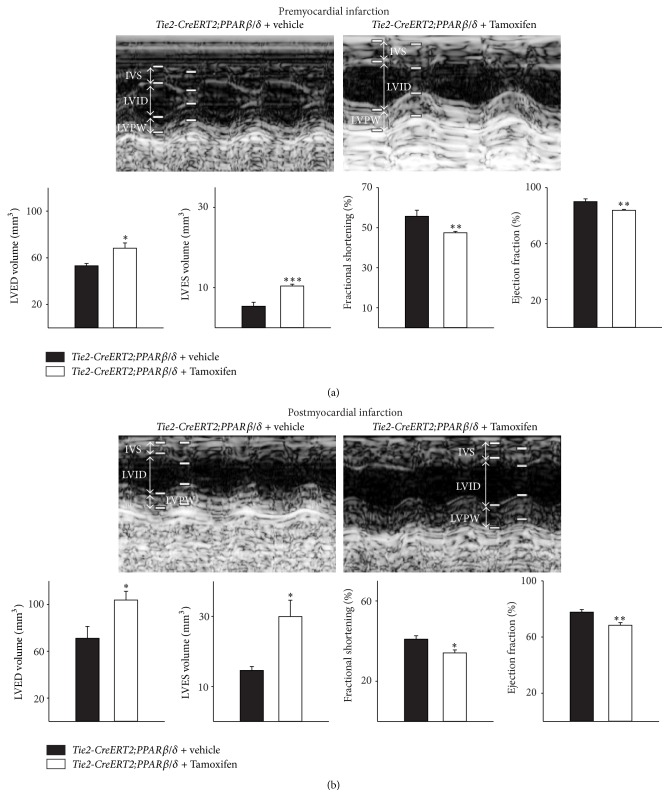
Impaired cardiac function upon vascular-specific PPAR*β*/*δ* overexpression, which worsens after myocardial infarction. (a) Echocardiographic examination indicates increased systolic and diastolic volumes, a reduced fractional shortening, and a decreased ejection fraction in animals with vessel-specific overexpression of PPAR*β*/*δ*, which becomes more evident after myocardial infarction (b) (*Tie2-CreERT2;PPARβ/δ* + Tamoxifen, *n* = 8, and* Tie2-CreERT2;PPARβ/δ* + vehicle, *n* = 5). IVS: interventricular septum; LVID: left ventricular internal diameter; LVPW: left ventricular posterior wall. Data are means ± SEM. ^*∗*^ *p* < 0.05, ^*∗∗*^ *p* < 0.01, and ^*∗∗∗*^ *p* < 0.001.

To test whether the angiogenesis induced cardiac hypertrophy affects cardiac function, we performed premyocardial and three-week postmyocardial infarction echocardiographic examinations in* Tie2-CreERT2;PPARβ/δ* animals induced with Tamoxifen and the respective controls treated with vehicle. Consistent with the observed cardiac hypertrophy, mice with vascular-specific overexpression of PPAR*β*/*δ* showed an increase in left ventricular end-diastolic (LVED) and -systolic (LVES) volume. Fractional shortening and the ejection fraction were slightly reduced as compared to the respective controls (Figure 6(a)).

Three weeks after myocardial infarction, control* Tie2-CreERT2;PPARβ/δ* animals treated with vehicle also showed an increase in the left ventricular end-diastolic and -systolic volume as well as a reduction in the fractional shortening and ejection fraction when compared to their healthy status before chronic ischemic heart disease. However, the situation was far worse in the mice with vascular-specific overexpression of PPAR*β*/*δ*; both LVED and LVES volume were highly increased and the fractional shortening and ejection fraction severely diminished (Figure 6(b)). Most studies attributed to PPAR*β*/*δ* a cardioprotective role, as* in vitro* and* in vivo* data suggested that PPAR*β*/*δ* inhibits cardiomyocyte apoptosis [26], protects against lipotoxicity [2], reduces cardiomyocyte hypertrophy [27], and, if overexpressed in cardiomyocytes, reduces myocardial injury due to ischemia/reperfusion [5]. Animals treated with PPAR*β*/*δ* agonists showed a rapid increase of the cardiac vasculature and an enhanced cardiac growth without functional impairment [6]. It seems as if the proper balance between PPAR*β*/*δ* activation in endothelial cells and cardiomyocytes (and maybe other cardiac cell types as fibroblasts) is required to confine the attribute “cardioprotective” to PPAR*β*/*δ*. Our results indicate that the specific, unbalanced activation of PPAR*β*/*δ* only in the vasculature, despite its effects on vessel and cardiac growth, is not sufficient to protect against chronic ischemic heart disease. Nevertheless, it is possible that activation of PPAR*β*/*δ* in the vasculature might have beneficial effects in the settings of smaller infarct sizes or in slowly developing arteriosclerosis, which will be subject of future studies.

## 4. Conclusions

In this study, we investigated the effects of a vascular-specific overexpression of PPAR*β*/*δ* on cardiac phenotype and function. The rapid induction of cardiac vessel formation was accompanied by an induction of cardiac growth, characterized by an increase in cardiomyocyte diameter. Upon myocardial infarction, the increased cardiac angiogenesis neither reduced infarct sizes nor improved the cardiac function. The proper balance of PPAR*β*/*δ* activation in the different cardiac cell types may be important for potential cardioprotective effects of PPAR*β*/*δ*.
