# Erratum to “Inducible Conditional Vascular-Specific Overexpression of Peroxisome Proliferator-Activated Receptor Beta/Delta Leads to Rapid Cardiac Hypertrophy”

**DOI:** 10.1155/2018/5480829

**Published:** 2018-10-29

**Authors:** Kay-Dietrich Wagner, Ana Vukolic, Delphine Baudouy, Jean-François Michiels, Nicole Wagner

**Affiliations:** ^1^Institute of Biology Valrose (iBV), University of Nice Sophia Antipolis, CNRS UMR7277/INSERM U1091, Faculty of Medicine, 06107 Nice, France; ^2^Institute for Molecular Health Sciences, ETH Zurich, 8093 Zurich, Switzerland; ^3^Department of Pathology, CHU Nice, 06002 Nice, France

In the article titled “Inducible Conditional Vascular-Specific Overexpression of Peroxisome Proliferator-Activated Receptor Beta/Delta Leads to Rapid Cardiac Hypertrophy” [1], there are errors in Figures 1, 2, and 5. In Figure 1, some of the images were not representative. A higher resolution figure is provided from the authors. In Figures 2 and 5, the images of hearts are supposed to be of different sizes. Due to production errors, the images were all the same size. The corrected figures are shown below.

## Figures and Tables

**Figure 1:**
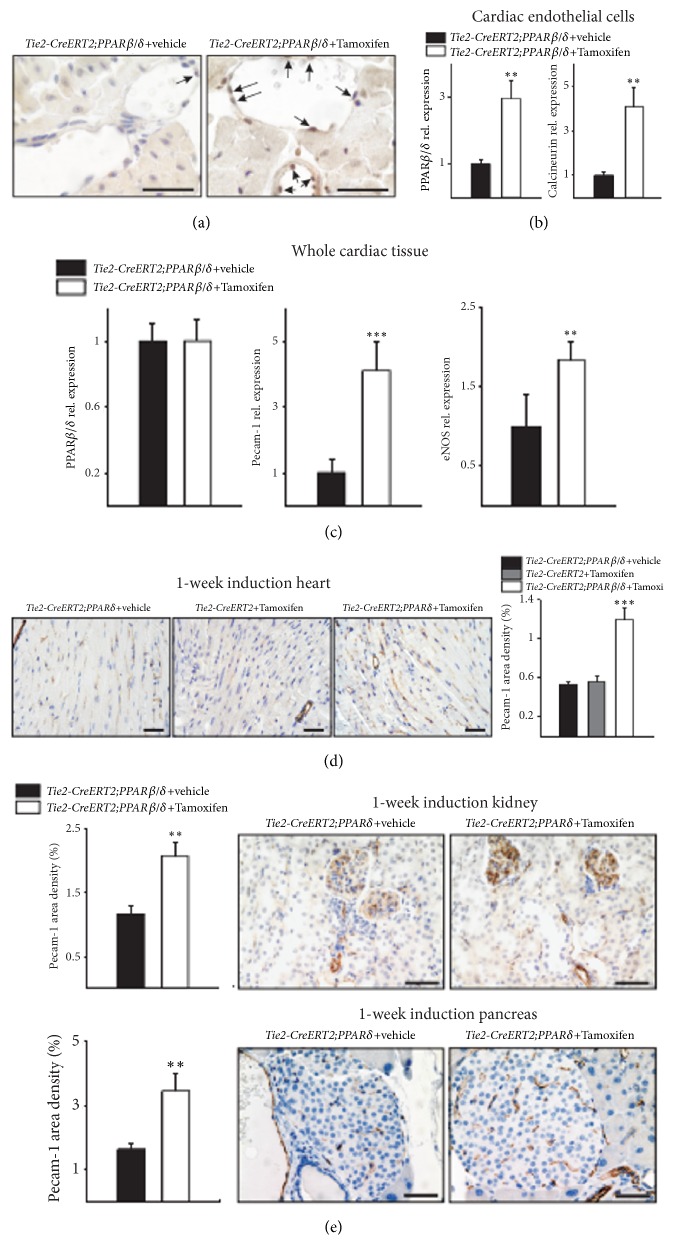


**Figure 2:**
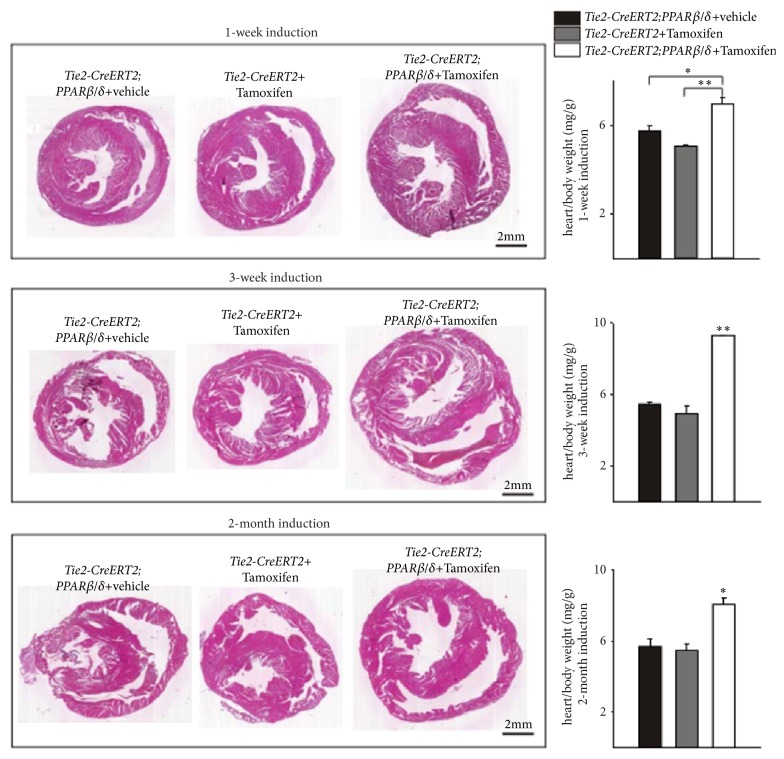


**Figure 5:**
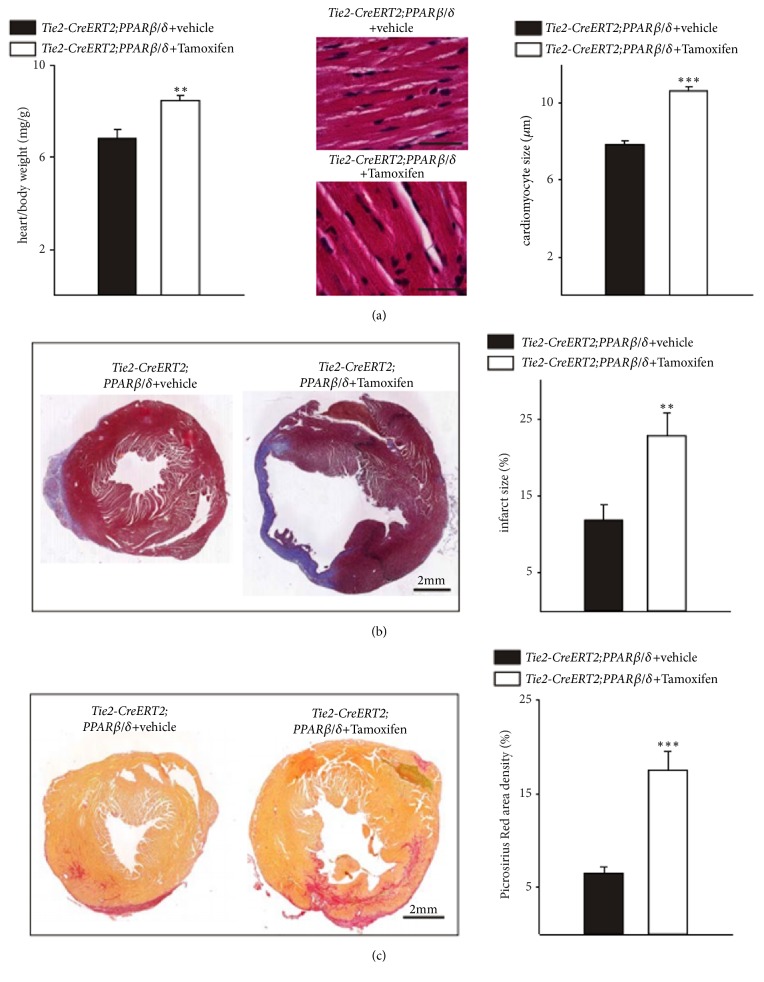


## References

[1] Wagner K.-D., Vukolic A., Baudouy D., Michiels J.-F., Wagner N. (2016). Inducible conditional vascular-specific overexpression of peroxisome proliferator-activated receptor beta/delta leads to rapid cardiac hypertrophy. *PPAR Research*.

